# Supplementary figures and images for: Evaluation of the articular cartilage in the knees of rats with induced arthritis treated with curcumin
Source: PLoS One. 2020 Mar 12;15(3):e0230228. doi: 10.1371/journal.pone.0230228 (PMC7067390; doi:10.1371/journal.pone.0230228)

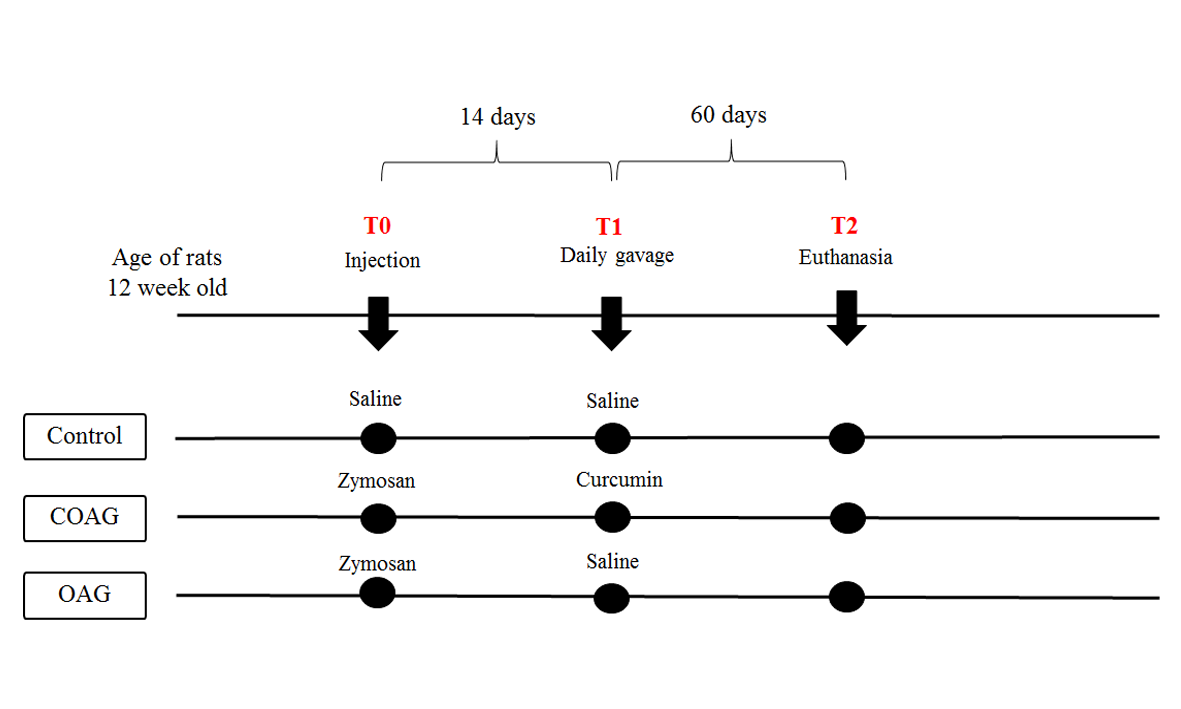

Supplement: S1 Fig — It demonstrates periods of rest, treatments and euthanasia. (TIF) [file pone.0230228.s001.tif]

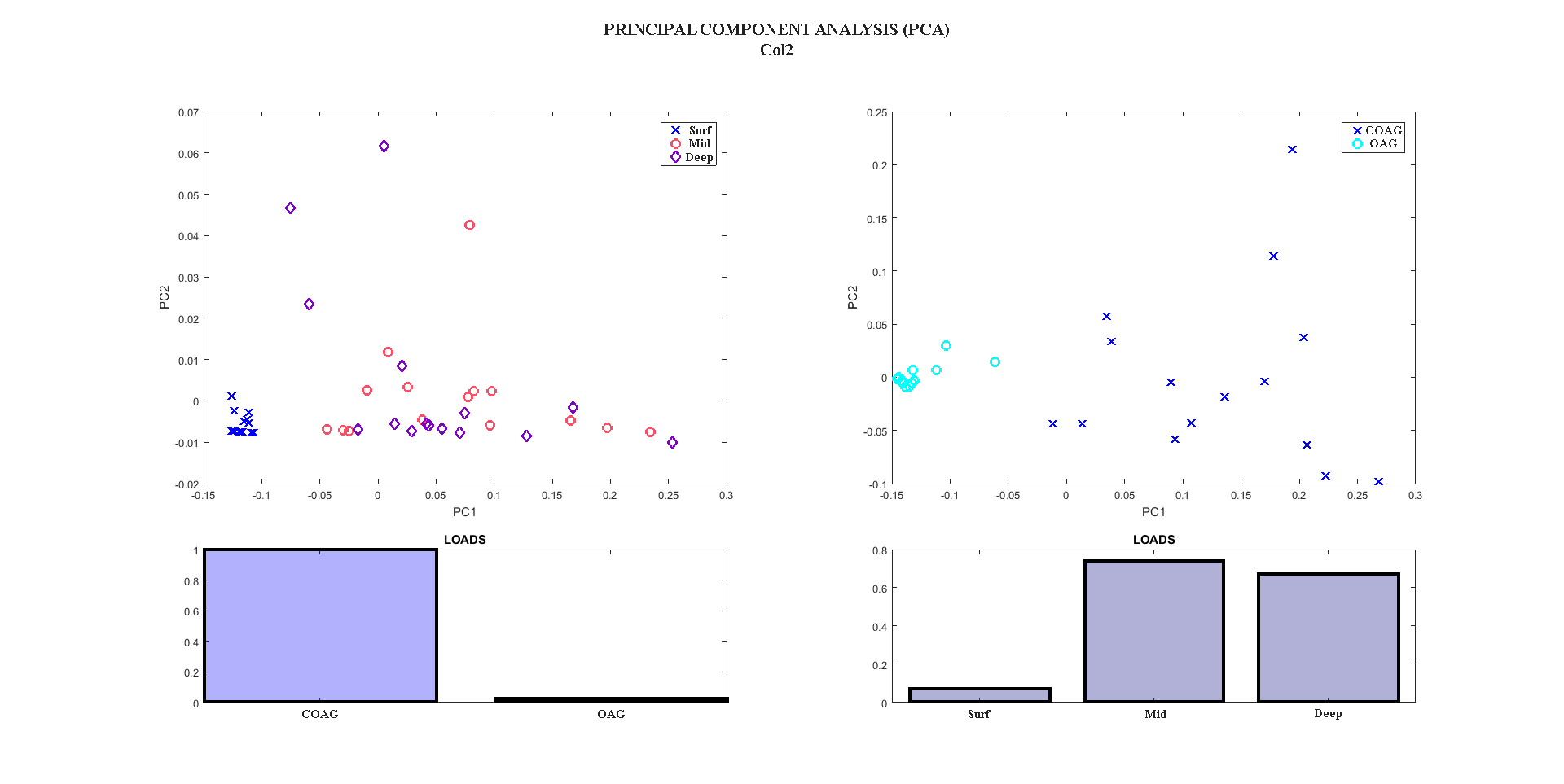

Supplement: S2 Fig — (A) The left upper quadrant shows the data distribution when considering the COAG and OAG as like variables and three cartilage layers as variables: surface (Surf), middle (Mid), and deep (Deep). The samples associated with the Surf cartilage layer displayed less variance and a high level of autosimilarity, while the Mid and Deep cartilage layers exhibited high variance and were indistinguishable. Moreover, the Surf sample cluster was linearly separated from the other clusters, emphasizing that the Surf factor had different statistical properties than the other factors. (B) The left lower quadrant shows the increased variance of the COAG variable in the PCA cluster. (C) The right upper quadrant shows the data distribution into the cartilage layers, Surf, Mid, and Deep, as variables of PCA and COAG and OAG as variable factors to evaluate the symmetry of the data. The samples from the OAG displayed less variance and a high level of autosimilarity, while the samples from the COAG exhibited a high level of variance. OAG and COAG clusters were also linearly separated, enforcing the statistically significant differences between these two groups. (D) The right lower quadrant show the high level of variance in the Mid and Deep variables in the PCA cluster, corroborating the findings from the inverse analysis. (TIF) [file pone.0230228.s002.tif]

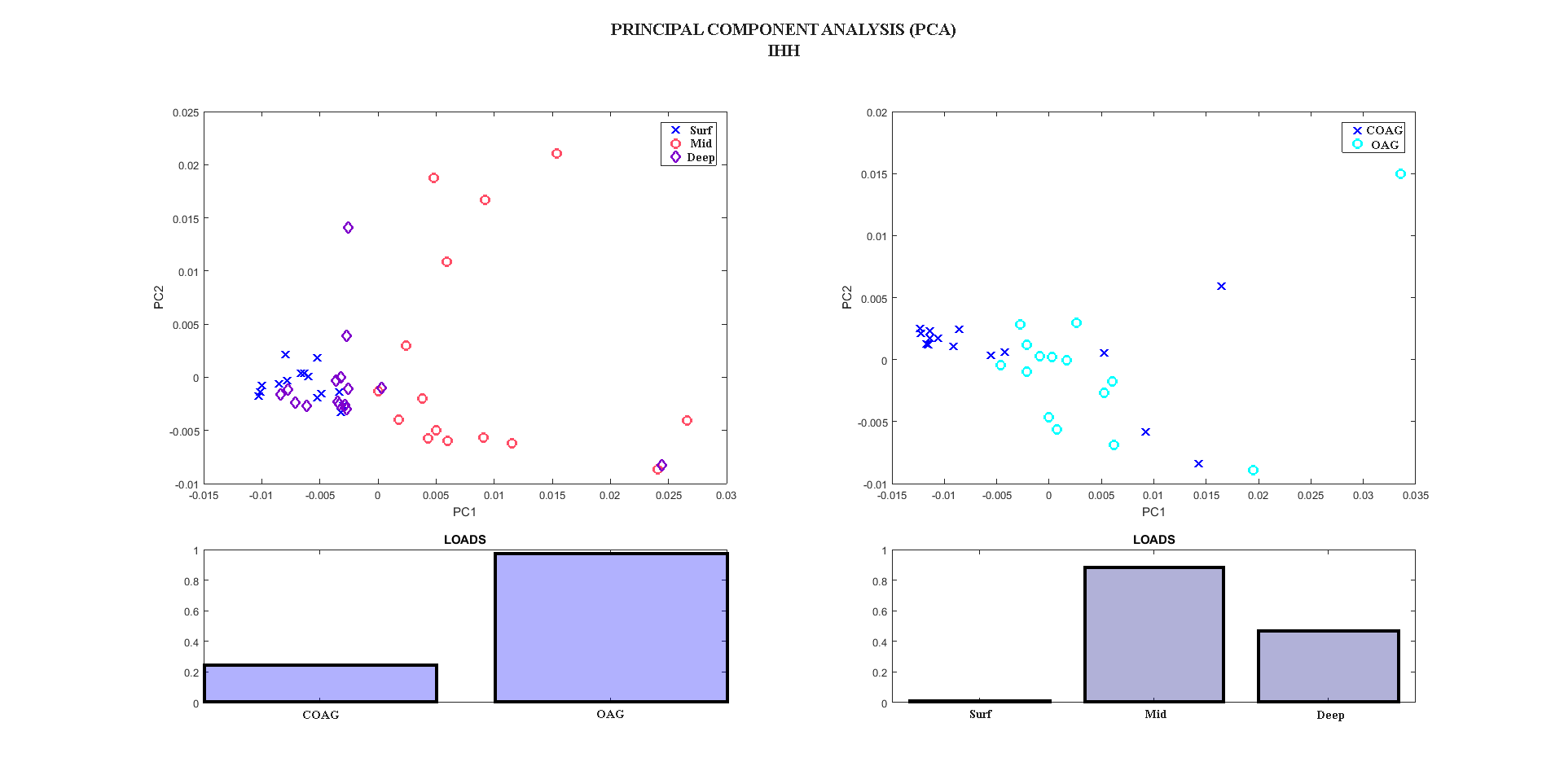

Supplement: S3 Fig — (A) The left upper quadrant shows the data distribution when considering the COAG and OAG as like variables and the three cartilage layers as variables: surface (Surf), middle (Mid), and deep (Deep). The samples associated with the Mid cartilage layer displayed a high level of variance and less autosimilarity, while Surf and Deep cartilage layers exhibited less variance and were indistinguishable. Moreover, the Mid sample cluster was linearly separated from the other factors, indicating a difference in statistical properties compared with the other factors. (B) The left lower quadrant shows the increased variance in the OAG variable in the PCA cluster. (C) The right upper quadrant shows the data distribution when considering the cartilage layers Surf, Mid, and Deep as variables of PCA and COAG and OAG as variable factors to evaluate the symmetry of the data. The samples from the COAG displayed less variance and a high level of autosimilarity, while the OAG samples exhibited a high level of variance. COAG and OAG clusters were not linearly separated, confirming the same statistical relationship between these two groups. (D) The right lower quadrant shows the high level of variance in the Mid variable in the PCA cluster, corroborating the results from the inverse analysis. (TIF) [file pone.0230228.s003.tif]

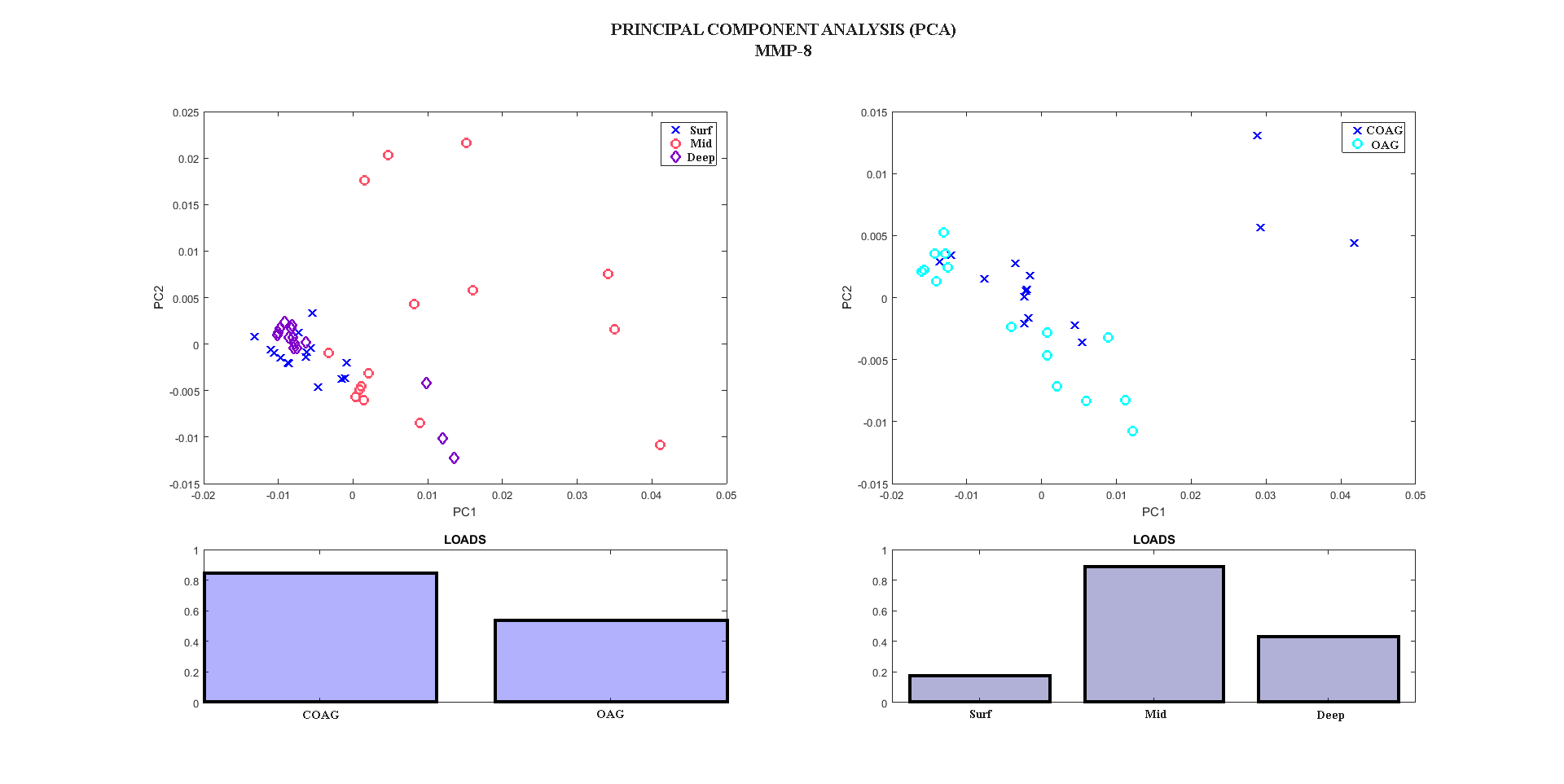

Supplement: S4 Fig — (A) The left upper quadrant shows the data distribution when considering the COAG and OAG as like variables and the three cartilage layers as variables: surface (Surf), middle (Mid), and deep (Deep). The samples associated with the Mid cartilage layer exhibited a high level of variance and less autosimilarity, while the Surf and Deep cartilage layers displayed less variance and were indistinguishable. Furthermore, the Mid sample cluster were not linearly separated from the other clusters, emphasizing that the Mid factor had same statistical properties as the other factors. (B) The left lower quadrant shows the increased variance of the COAG variable in the PCA cluster. (C) The right upper quadrant shows the data distribution when considering the cartilage layers Surf, Mid, and Deep as variables in PCA and COAG and OAG as variable factors to evaluate the symmetry of the data. The samples from the OAG displayed less variance and were indistinguishable from the COAG. The OAG and COAG clusters were also not linearly separated, confirming the same statistical relationship between these two groups. (D) The right lower quadrant shows the high level of variance of the Mid variable, followed by Deep and Surf in the PCA cluster, corroborating the results of the inverse analysis. (TIF) [file pone.0230228.s004.tif]

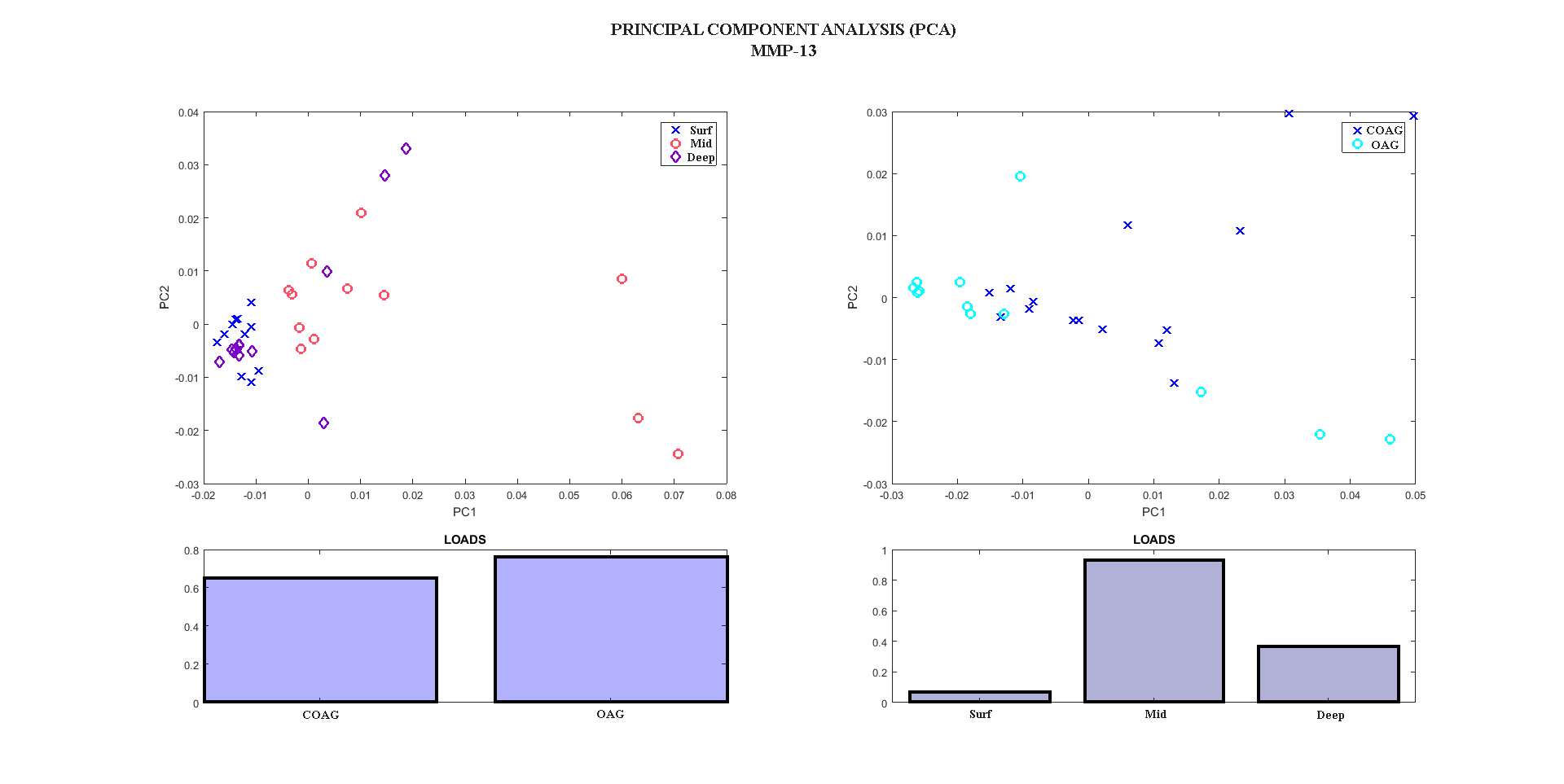

Supplement: S5 Fig — (A) The left upper quadrant shows the distribution of data when considering the groups COAG and OAG as like variables and the three cartilage layers as variables: surface (Surf), middle (Mid), and deep (Deep). The samples associated with the Mid cartilage layer displayed a high level of variance and less autosimilarity, while Surf and Deep cartilage layers displayed less variance and were indistinguishable. Moreover, the Mid sample cluster were not linearly separated from the other clusters, indicating that the Mid factor had the same statistic as the other factors. (B) The left lower quadrant shows the increased variance of the OAG variable in the PCA cluster. (C) The right upper quadrant shows the data distribution when considering the cartilage layers Surf, Mid, and Deep as variables of PCA and COAG and OAG as variable factors to evaluate the symmetry of the data. The samples from the COAG displayed less variance and more autosimilarity, while the OAG samples displayed a high level of variance. COAG and OAG clusters were also not linearly separated, suggesting the same statistical relationship between these two groups. (D) The right lower quadrant shows the high level of variance of the Mid variable in the PCA cluster, corroborating the results of the inverse analysis. (TIF) [file pone.0230228.s005.tif]

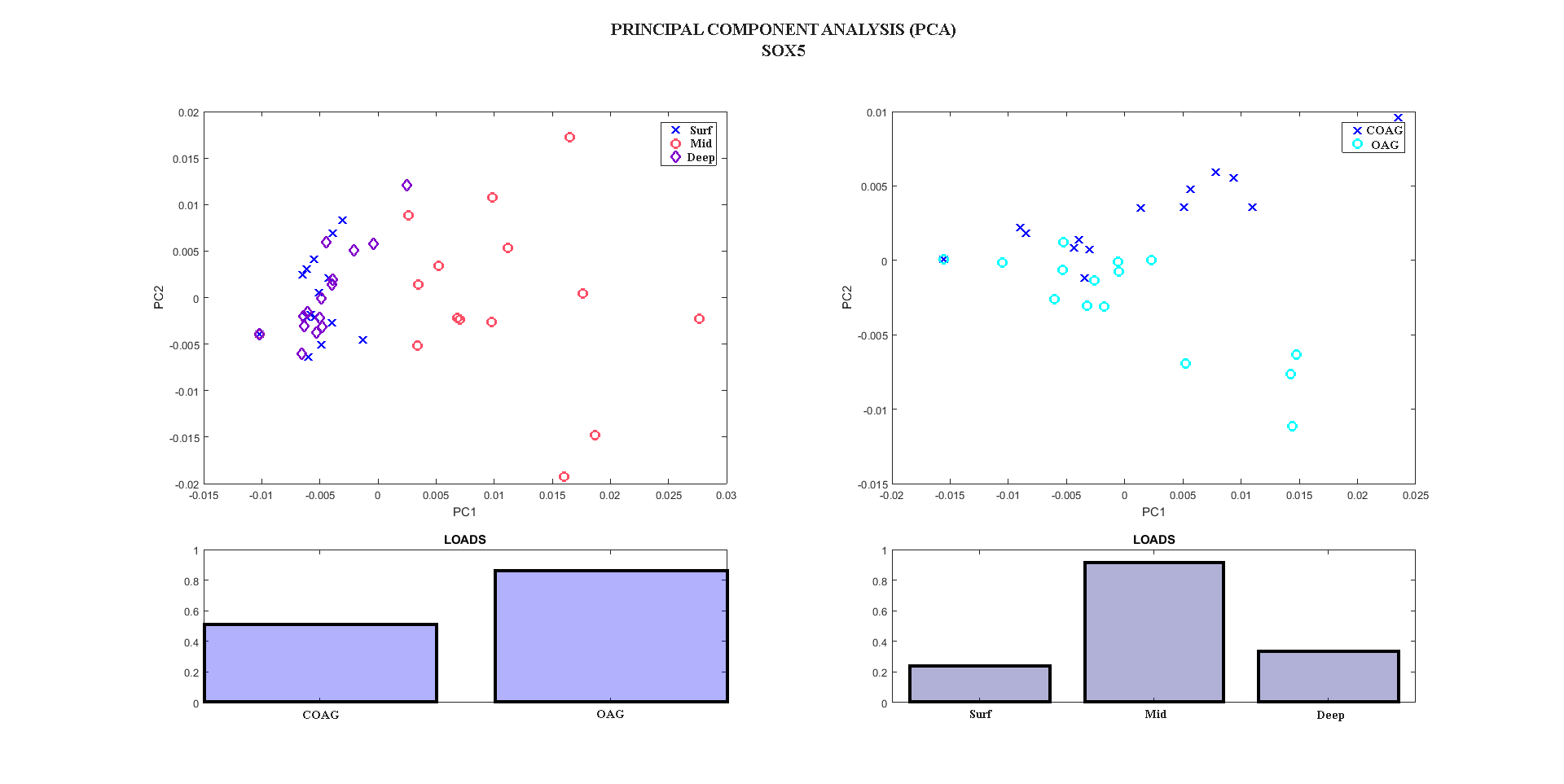

Supplement: S6 Fig — (A) The left upper quadrant shows the data distribution when considering the COAG and OAG as like variables and the three cartilage layers as variables: surface (Surf), middle (Mid), and deep (Deep). The samples associated with the Mid cartilage layer displayed a high level of variance and less autosimilarity, while Surf and Deep cartilage layers exhibited less variance and were indistinguishable. Furthermore, the Mid samples were linearly separated from the other clusters, emphasizing that the Mid factor had different statistics than the other factors. (B) The right lower quadrant showed the high level of variance in the OAG variable in the PCA cluster. (C) The right upper quadrant shows the data distribution when considering the cartilage layers, Surf, Mid, and Deep, as variables in PCA and COAG and OAG as variable factors to evaluate the symmetry of the data. The samples from the COAG displayed less variance and a high level of autosimilarity, while the samples from the OAG had a high level of variance. The COAG and OAG clusters were not linearly separated, revealing the same statistical relationship between these two groups. (D) The right lower quadrant shows the high level of variance in the Mid variable in the PCA cluster, corroborating the results of the inverse analysis. (TIF) [file pone.0230228.s006.tif]

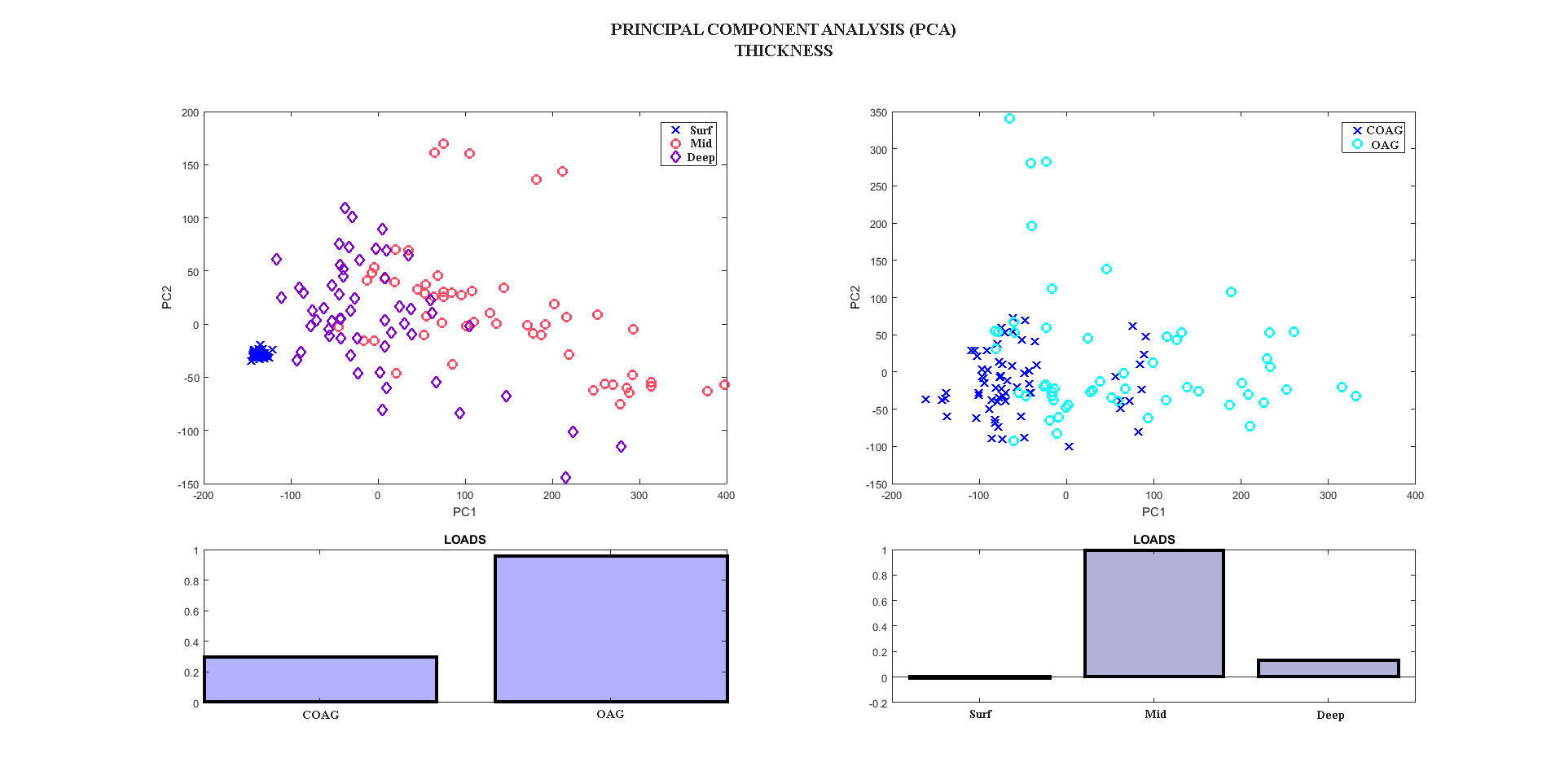

Supplement: S7 Fig — (A) The left upper quadrant shows the data distribution when considering the COAG and OAG as like variables and the three cartilage layers as variables: surface (Surf), middle (Mid), and deep (Deep). The samples associated with the Surf cartilage layer displayed less variance and a high level of autosimilarity, while the Deep cartilage layer displayed a high level of variance and was indistinguishable from the Mid layer. Furthermore, the Surf sample cluster were linearly separated from the other clusters, emphasizing that the Surf factor had different statistical properties from the other factors. (B) The left lower quadrant shows the increased variance of the OAG variable in the PCA cluster. (C) The right upper quadrant shows the data distribution when considering the cartilage layers Surf, Mid, and Deep as variables of PCA and COAG and OAG as variable factors to evaluate the symmetry of the data. The samples from the COAG displayed less variance and were indistinguishable from the OAG. COAG and OAG clusters were not linearly separated, confirming the same statistical relationship between these two factors. (D) The right lower quadrant shows the high level of variance of the Mid variable, followed by the Deep and Surf variables in the PCA cluster, corroborating the results of the inverse analysis. (TIF) [file pone.0230228.s007.tif]

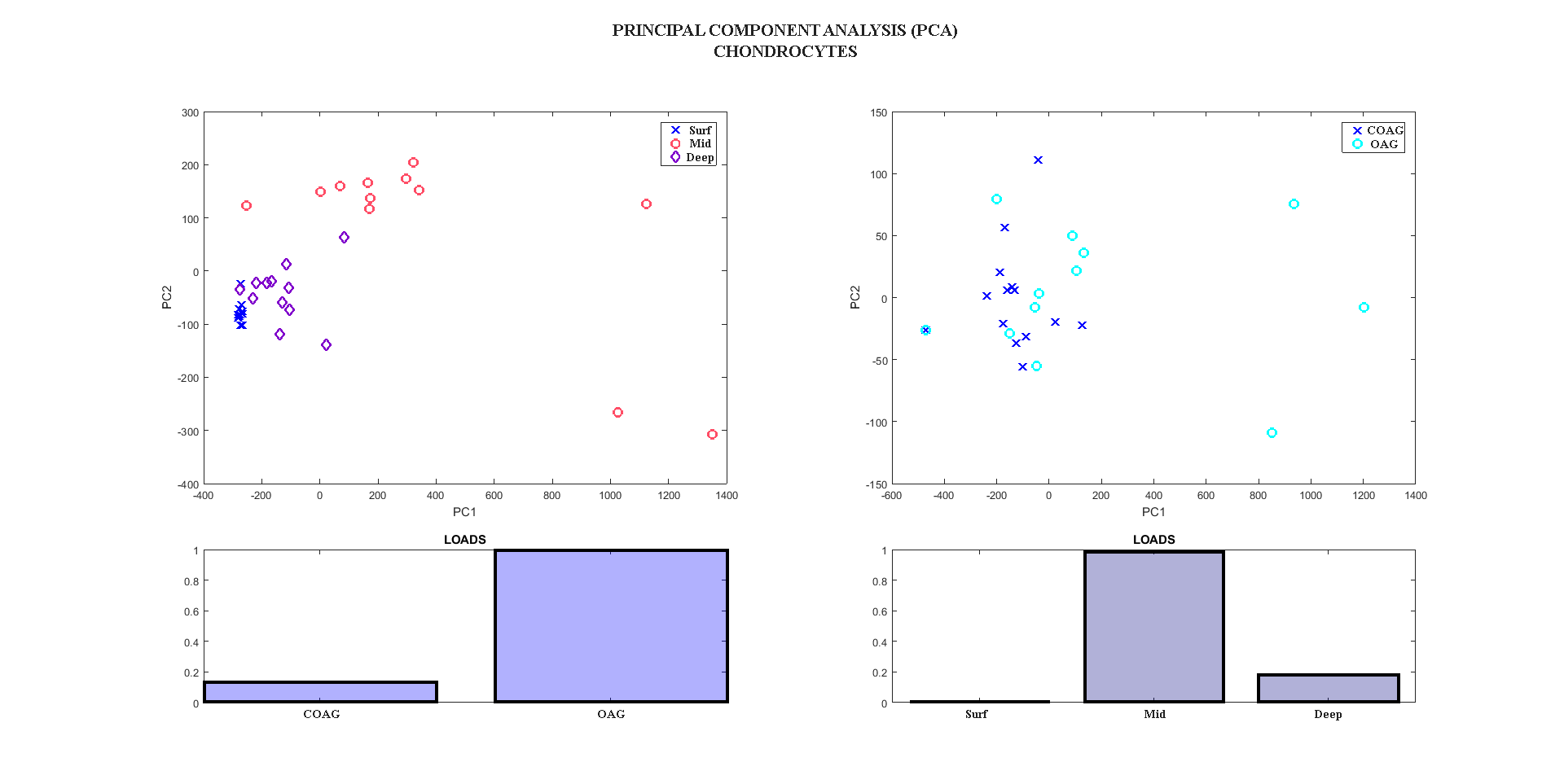

Supplement: S8 Fig — (A) The left upper quadrant shows the data distribution when considering the COAG and OAG as like variables and the three cartilage layers as variables: surface (Surf), middle (Mid), and deep (Deep). The samples associated with the Surf cartilage layer displayed less variance and a high level of autosimilarity, while the Deep cartilage layer showed less variance and a high level of autosimilarity compared with those in the Middle cartilage layer. Moreover, the Surf, Mid, and Deep sample clusters were linearly separated from the other clusters, emphasizing that the Surf, Mid, and Deep factors possessed different statistical properties than the other factors. (B) The left lower quadrant shows the increased variance of the OAG variable in the PCA cluster. (C) The right upper quadrant shows the data distribution when considering the cartilage layers Surf, Mid, and Deep as variables of PCA and COAG and OAG as variable factors to evaluate the symmetry of the data. The samples from the COAG displayed less variance and were indistinguishable from the OAG. COAG and OAG clusters were not linearly separated, confirming the same statistical relationship between these two factors. (D) The right lower quadrant shows the high level of variance of the Mid variable, followed by the Deep and Surf variables in the PCA cluster, corroborating the results of the inverse analysis. (TIF) [file pone.0230228.s008.tif]
