# Supplementary material for: Evaluation of the articular cartilage in the knees of rats with induced arthritis treated with curcumin
Source: PLoS One. 2020 Mar 12;15(3):e0230228. doi: 10.1371/journal.pone.0230228 (PMC7067390; doi:10.1371/journal.pone.0230228)
Supplement: Attachment — Submitted filename: Response to the reviewers.docx [file pone.0230228.s009.docx]

Response to the reviewers

First of all, we'd like to inform that all changes in order to meet your comments are highlighted in yellow in this revised version of the manuscript. Suggestions that could not be performed were justified.

Journal Requirements-

1. Please include the following information relating to animal experiments in your Methods section: The frequency of animal monitoring, including the specific criteria you used to monitor animal health.

Authors reply: The requested data was inserted in the text: “The animals were daily monitored for abscesses and amount of food and water consumption”.

2. Please also justify the chosen dose of curcumin administered to the animals.

Authors reply: The administered dose was based on previous studies: doi:10.1186/s13075-016-1025-y. The manuscript was inserted in the text and references.

3. We noticed you have some minor occurrence of overlapping text with the following previous publication(s), which needs to be addressed:

<https://doi.org/10.1007/s00441-013-1658-y>.

In your revision ensure you cite all your sources (including your own works), and quote or rephrase any duplicated text outside the Methods section. Further consideration is dependent on these concerns being addressed.

Authors reply: The part of methods section in which there was occurrence of duplicated text with ours previous manuscripts was changed and this manuscript was also cited in the references.

4. We suggest you thoroughly copyedit your manuscript for language usage, spelling, and grammar. If you do not know anyone who can help you do this, you may wish to consider employing a professional scientific editing service.

Authors reply: The manuscript was edited for proper English language, grammar, punctuation, spelling, and overall style by one or more of the highly qualified native English speaking editors at American Journal Experts.

The certificate was issued on January 20, 2020 and may be verified on the AJE website using the verification code EB79-E1ED-0019-06E7-3719.

5. PLOS requires an ORCID iD for the corresponding author in Editorial Manager on papers submitted after December 6th, 2016.

Authors reply: The Orcid iD was associated for the Corresponding author.

Reviewer 1

“Evaluation of rat knee articular cartilage with induced arthritis and treated with Curcumin” by Tiago Nicoliche et al. attempts to evaluate the anti-inflammatory effects of curcumin in the induced osteoarthritis treatment in rats’ knee. I recognize the effort made or the authors to develop this histologic descriptive study about some keys elements of OA development. Conceptually this manuscript is very similar to other manuscripts published (Mol Med Rep. 2017 Aug;16(29); Osteoarthritis Cartilage. 2015 Jan;23(1):94-102; Oxid Med Cell Longev. 2019 May 16;2019; Biosci Rep. 2018 Jul 2;38(4); Mol Med Rep. 2017 Aug;16(29). So, what is the novelty of this study? I do not think that this manuscript adds significantly to what is already know and is not novel enough to warrant a new full length publication. I have the following suggestions for improvement.

Authors reply: First of all, thank you for the additional manuscripts concerning curcumin studies, all of them were inserted in the text and reinforced the medical properties of curcumin for future human routine use.

Moreover, our study works with the induction of osteoarthritis by zymosan, a different technique from those presented in the manuscripts above (destabilization of the medial meniscus, surgical transection of the right anterior cruciate ligament, natural aging, the use of pristane, chondrocyte culture isolated from rats), this demonstrates that curcumin has protective effects on OA articular cartilage in several OA induction techniques.

In the treatment of our study, the animals were treated by gavage, while other procedures were performed by Feng et al. (intraperitoneal injection), Horcajada et al. (diet based on curcumin) and Wang et al. (chondrocyte culture). On the other hand, Zhang et al. used the gavage route for treatment, but animal models were natural aging and destabilization of the medial meniscus to induce AO.

Our work evaluated the proliferation of chondrocytes, the expression of IHH, SOX-5, Col2, MMPs 8 and 13 in each layer of the articular cartilage (superficial, intermediate and deep) and measured the thickness of each layer of the cartilage articulate. The manuscripts mentioned above or any other in the curcumin literature investigated all these factors and made cartilage morphometry.

1) The style of the manuscript should be improved. The discussion reads like a review at certain places. It should be more focused, discussing the experimental findings in light of the available data in the literature as they relate to the development of osteoarthritis. “In studies carried out by Fernandes et al. … Burrage et al. (2006) believed that … Tetlow et al. (2001) (40) found …” This paragraph reads like a list without concluding what is unknown, for example.

Authors reply: The discussion was redesigned as requested.

2) As it is suggested that curcumin could be administered as part of a dietary supplementation aimed at preventing inflammation and reducing OA severity, it is important to fully analyse the effects of the tested dose on control animals in addition to those subjected to the OA model. Does orally administered curcumin also improved Col2, IHH, SOX-5 expressions and the number of chondrocytes in unchallenged joints or in naturally ageing animals?

Authors reply: Unfortunately, this group was not inserted in the study but previous studies have shown that curcumin inhibits nuclear factor κB‐mediated inflammatory signaling (NF-KB) in many cell types, including chondrocytes (doi.org/10.1111/j.1749-6632.2009.04687.x). As NF-KB is increased in a variety of tissues with aging and is associated with numerous age-related degenerative diseases (Tilstra JS, Clauson CL, Niedernhofer LJ, Robbins PD. NF-κB in Aging and Disease. Aging Dis. 2011;2(6):449–465.), we may conclude that these factors may also be improved in unchallenged joints and in ageing. Anyway, the manuscript (doi:10.1042/BSR20171691 – which was sent by the revisor) was inserted in the discussion.

3) Does the curcumin lead to any reduction in synovial tissue inflammation? At least the presentation of wider field images of the joint including synovial and cartilage would help in understanding the therapeutic impact of the treatment.

Authors reply: Unfortunately, we do not have wider field of the specimens as soft tissue was dissected. But, according to previous studies on synovial inflammation (doi:10.1016/j.joca.2014.08.016.), the group treated with rutin and curcumin compared to the treated with rutin, did not shown additional effects on histological or synovial results.

4) Please provide more specific reasons why intraarticular injection of Zymosan is a proper model of OA, since the mechanism of OA is not fully understood.

Authors reply: According to Asquith et al. (2009), doi:10.1002/eji.200939578, there are several models to induce OA. These models include the collagen-induced arthritis, collagen-antibody-induced arthritis, zymosan-induced arthritis, and the methylated BSA model, and the genetically manipulated or spontaneous arthritis models such as the TNF-alpha-transgenic mouse, K/BxN mouse, and the Skg mouse. All these methods are widely used but the zymosan binds to TLR2 in macrophages leading to the induction of proinflammatory cytokines, arachidonate mobilization, protein phosphorylation and also activates complement via the alternative pathway. The intra‐articular injection model precludes analysis of the systemic component of the disease but it was not our focus in this study.

5) Is there clinical report or field study showing that people regularly consuming diet containing curcumin have lower rate of OA compared to those not consuming such diet?

Authors reply: Regular consumption of curcumin has already shown improvements in human OA. In the study of Belcaro et al. (2010), Meriva, a proprietary curcumin-phosphatidylcholine phytosome complex, treatment was investigated in a longer (eight months) study involving 100 OA patients and resulted in significant improvement of both the clinical and biochemical end points scores.

Minor comments:

1) The Schedule of OA induction and treatment it is not clear. Animals were euthanized 8 weeks after induction of OA? or 8 weeks after curcumin was daily administered for 60 days?

Authors reply: It was inserted a new supplementary figure (S1 Fig) to elucidate the studied groups, periods of treatment and euthanasia.

2) The results should be described in the same order than results are presented in figures.

Authors reply: The suggestion was accepted and the order of the figures was altered in accordance to the text.

3) A figure describing possible model for how curcumin modulate inflammation could be good.

Authors reply: It was inserted a new figure (Fig 1. Diagrammatic representation of the curcumin role on inflammation and Col2 degradation.) in accordance to the requested by the reviewer.
